# Preliminary Analysis of the Expression of Selected Proangiogenic and Antioxidant Genes and MicroRNAs in Patients with Non-Muscle-Invasive Bladder Cancer

**DOI:** 10.3390/jcm5030029

**Published:** 2016-02-25

**Authors:** Magdalena Kozakowska, Barbara Dobrowolska-Glazar, Krzysztof Okoń, Alicja Józkowicz, Zygmunt Dobrowolski, Józef Dulak

**Affiliations:** 1 Department of Medical Biotechnology, Faculty of Biochemistry, Biophysics and Biotechnology, Jagiellonian University, 30-387 Krakow, Poland; m.kozakowska@uj.edu.pl (M.K.); alicja.jozkowicz@uj.edu.pl (A.J.); 2 Department of Pediatric Urology, Jagiellonian University Medical College, 30-688 Krakow, Poland; bdobrowolska@o2.pl; 3 Department and Clinic of Urology, Jagiellonian University Medical College, 31-530 Krakow, Poland; zdobrowol@su.krakow.pl; 4 Department of Pathomorphology, Jagiellonian University Medical College, 31-531 Krakow, Poland; k.okon@uj.edu.pl; 5 Department of Urology, University of Rzeszow, 35-959 Rzeszow, Poland; 6 Malopolska Centre of Biotechnology, Jagiellonian University, 30-387 Krakow, Poland

**Keywords:** bladder cancer, urothelial cancer, heme oxygenase-1, hypoxia inducible factor, Nrf2, miR-155, miR-200c, VEGF, angiogenesis

## Abstract

Heme oxygenase-1 (HO-1) is an enzyme contributing to the development and progression of different cancer types. HO-1 plays a role in pathological angiogenesis in bladder cancer and contributes to the resistance of this cancer to therapy. It also regulates the expression of microRNAs in *rhabdomyosarcoma* and non-small cell lung cancer. The expression of HO-1 may be regulated by hypoxia inducible factors (HIFs) and Nrf2 transcription factor. The expression of HO-1 has not so far been examined in relation to Nrf2, HIF-1α, and potential mediators of angiogenesis in human bladder cancer. We measured the concentration of proinflammatory and proangiogenic cytokines and the expression of cytoprotective and proangiogenic mRNAs and miRNAs in healthy subjects and patients with bladder cancer. HO-1 expression was upregulated together with HIF-1α, HIF-2α, and Nrf2 in bladder cancer in comparison to healthy tissue. VEGF was elevated both at mRNA and protein level in the tumor and in sera, respectively. Additionally, IL-6 and IL-8 were increased in sera of patients affected with urothelial bladder cancer. Moreover, miR-155 was downregulated whereas miR-200c was elevated in cancer biopsies in comparison to healthy tissue. The results indicate that the increased expression of HO-1 in bladder cancer is paralleled by changes in the expression of other potentially interacting genes, like Nrf2, HIF-1α, HIF-2α, IL-6, IL-8, and VEGF. Further studies are necessary to also elucidate the potential links with miR-155 and miR-200c.

## 1. Introduction

Bladder cancer (*urothelial cancer*) is the 7th most common cancer in men and 17th in women, and is more frequent in well-developed regions, where 60% of all incidents occur. Non-muscle-invasive bladder cancer is characterized by a high rate of recurrence—despite the total resection of the tumor it reappears in 75% of patients. The five-year survival rate is around 57% [1,2]. The major cause of development of bladder cancer is long-term exposure to environmental risk factors. The primary culprits are smoking, chemical compounds binding DNA (like aromatic amines), or arsenic (the metabolism of which is associated with the generation of reactive oxygen species) [2]. Recent data indicate the role of oxidative stress in the progression of bladder cancer [3].

Among transcription factors affected by oxidative stress, and which are altered in bladder cancer, are hypoxia inducible factors (HIF-1α, HIF-2α), and Nrf2 transcription factor [3]. In response to oxidative stress, Nrf2 binds to promoters of genes encoding antioxidative enzymes [4]. It is believed to be a mediator of action of chemopreventive compounds [5,6,7,8], and it also contributes to resistance to cisplatin [9] and photodynamic therapy [10]. HIF-1α and HIF-2α, which regulate cellular redox homeostasis, are factors inducing angiogenesis and inflammatory reaction [11,12]. They are correlated with increasing invasiveness, macrophage infiltration, and angiogenesis in bladder cancer [13,14,15]. Among the direct mediators of HIFs in bladder cancer, vascular endothelial growth factor (VEGF) is usually listed [13,15,16,17]. Its expression correlates with enhanced angiogenesis, proliferation, and metastatic potential in urothelial tumors [18,19,20]. However, since clinical trials based on VEGF-targeted anti-angiogenic therapies of bladder cancer have not given satisfactory results [21], there is a need to search for other mediators of both pro-angiogenic and anti-cytotoxic effects of HIFs and Nrf2.

Heme oxygenase-1 (HO-1) is a heme-degrading enzyme of known pro-angiogenic and cytoprotective effects, the expression of which may be induced by both Nrf2 and HIF-1α [22]. Moreover, HO-1 has a potent impact on the development of different types of cancer [23]. In recent years, an increasing body of evidence points to the role of HO-1 in pathological angiogenesis in bladder cancer [24] and in some cases in the resistance of this cancer to chemo- and radiotherapy [10,25,26]. However, only a few of the studies analyzed clinical material from patients affected by bladder cancer, confirming a positive correlation of HO-1 level with the proliferation of cancer cells, VEGF-induced angiogenesis, and, finally, the malignant behavior of the cancer [24,27,28,29], whereas none of them involved comparison to the healthy tissue. Furthermore, the expression of HO-1 was never assessed together with Nrf2 in the clinical samples and only one study showed the analysis of HO-1 with HIF-1α and HIF-2α in urothelial tumors, suggesting the correlation between their expressions [24].

HO-1 is known to potently regulate the expression of miRNAs in muscle myoblasts and rhabdomyosarcoma [30,31,32]. The expression of miRNAs is also changed in bladder cancer, which may be a diagnostic parameter [33,34]. Changes in miR-200c are suggested to be associated with the pathogenesis of bladder cancer and to affect the efficacy of therapeutic treatment [35,36]. Similar tendencies were demonstrated for other types of cancer [37,38], but the current data for bladder cancer are ambiguous and show either an induction of miR-200c expression [39,40,41] or an inhibition [33,42,43,44,45]. On the other hand, miR-133b [46,47,48] and miR-133a [39,46,49,50,51,52], shown by us to be strongly affected by HO-1 [30], are also downregulated in bladder cancer.

The aim of this study was to analyze the level of proinflammatory and proangiogenic cytokines in the sera and determine the expression of genes associated with cytoprotection and angiogenesis as well as selected miRNAs in clinical samples collected from patients subjected to diagnostic and control cystoscopy.

## 2. Experimental Section

### 2.1. Patient Samples

Patients with known bladder cancer in stages Ta, Tis, or T1 and patients with suspected bladder cancer were recruited (*N* = 21; age 51–80, mean age = 67; five females and 16 males). Two hours prior to the TURBT (transurethral resection of the bladder tumor) procedure, patients underwent bladder instillation with 50 mL of 8 mM solution of HAL (hexyl aminolevulinate) hydrochloride in phosphate buffered saline (Hexvix, Photocure) through a Foley catheter. After the HAL solution was evacuated, the bladder was inspected by white light cystoscopy. Lesions or suspicious areas were classified and mapped onto a bladder chart in blue. The bladder was then inspected by HAL fluorescence cystoscopy. Lesions or suspicious areas were classified and mapped onto the bladder chart in red. Fluorescence cystoscopy was a supplementary but not substitutional procedure. The diameter of the lesions or suspicious areas were 0.2–2 cm, while the majority did not exceed 1 cm. Biopsies (0.1–0.3 cm diameter) were taken from all mapped areas. Test materials were collected for histopathological analysis and some of them were used for the isolation of RNA. Among 27 samples collected for mRNA and miRNA analysis, papillary urothelial neoplasm of low malignant potential, according to the International Society of Urological Pathology guidelines [53], was diagnosed in two cases, low-grade urothelial carcinoma in 13, and high-grade urothelial carcinoma in one (out of the initial group of 21 patients, bladder cancer was diagnosed in *N* = 16 cases; age 51–80, mean age 67; five females and 11 males). Eleven samples for mRNA and miRNA analysis were histologically assessed as unaltered, healthy tissue—*N* = 11, age 57–74, mean age 67; two females and nine males. Those 11 samples were derived from patients finally diagnosed as healthy (*N* = 5, age 58–73, mean age 69; 5 males) and 6 samples of healthy tissue were also found among patients who had bladder cancer confirmed in another area.

Serum was collected for the analysis of cytokines from all patients subjected to cystoscopy (16 patients with subsequently diagnosed bladder cancer and five assessed histopathologically as healthy) as well as from additional healthy, voluntary, age-matched controls (the total number of healthy controls included for the measurement of cytokine: *N* = 9, age 51–73, mean age = 65; three females and six males).

The research was completed in September 2012; it complied with the Declaration of Helsinki and was approved by the Local Bioethical Commission (agreement No. KBET/197/B/2012). Patients provided written informed consent for the study.

### 2.2. RNA Isolation and qRT-PCR

RNA isolation followed by reverse transcription and quantitative PCR for genes and miRNA were performed with standard procedures, described elsewhere [30]. Primers used in qRT-PCR are presented in Table 1 and Table 2.

### 2.3. Luminex Analysis of Cytokine and Growth Factor Concentrations in Plasma

Concentrations of interferon-γ (IFN-γ), interleukin (IL)-1β, IL-6, IL-8, IL-10, IL-12, IL-17, monocyte chemoattractant protein-1 (MCP-1), tumor necrosis factor-α (TNFα), and VEGF in plasma were evaluated using Milliplex FlexMap 3D (Millipore, Billerica, MA, USA) according to the vendor’s protocol.

### 2.4. Statistical Analysis

The normal distribution of data was checked using the D’Agostino–Pearson test. Statistical significance was assessed using the Student’s t-test or Welch’s Mann–Whitney *U*-test, and accepted at *p* < 0.05. Correlation was analyzed using Spearman’s rank correlation.

## 3. Results

### 3.1. Level of Cytokine in the Sera

**Figure 1:**
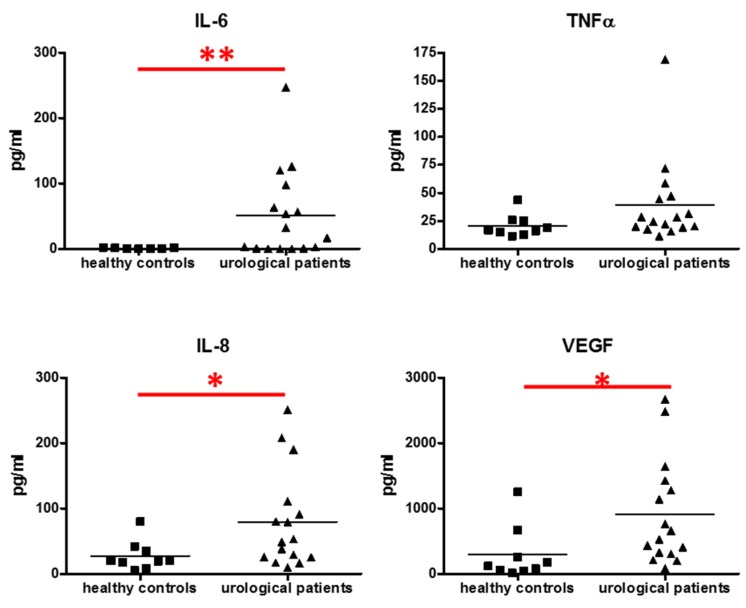
Concentrations of cytokines in the serum of patients with diagnosed bladder cancer and in healthy controls. Luminex, *N* = 9–16; each dot represents one individual, line represents a mean; * *p* < 0.05; ** *p* < 0.01.

The analysis of cytokine levels was performed in the sera of patients with diagnosed bladder cancer (*N* = 16) and aged-matched healthy controls (*N* = 9). IL-6 was significantly increased in the material collected from patients affected by bladder cancer, whereas TNFα showed a tendency to be induced (*p* = 0.08) (Figure 1). Proangiogenic VEGF and IL-8 were both significantly increased in urological patients (Figure 1), whereas IFNγ, IL-1β, MCP-1, IL-10, IL-12, and IL-17 were unchanged (data not shown).

### 3.2. Expression of Proangiogenic and Cytoprotective Genes in Tumor Samples

**Figure 2:**
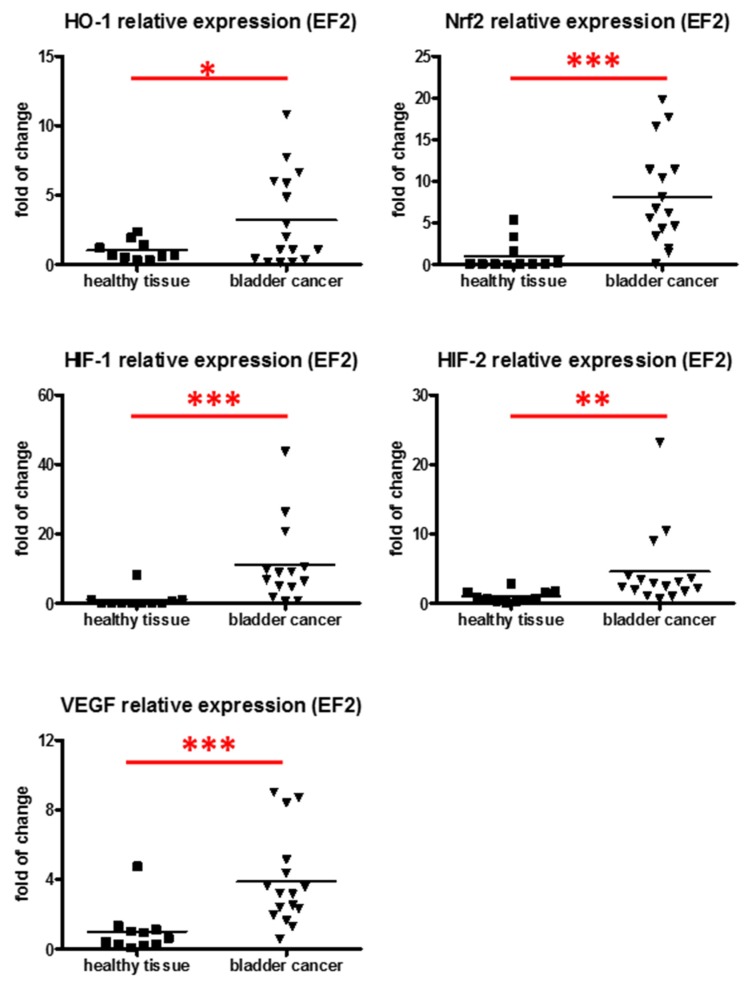
Gene expression at mRNA level in samples of bladder cancer and in healthy tissue. qRT-PCR, *N* = 11–16; each dot represents one individual, line represents a mean; * *p* < 0.05; ** *p* < 0.01; *** *p* < 0.001.

The analysis of gene expressions at mRNA level revealed that Nrf2 (a transcription factor and regulator of the expression of proteins that are a second line of cell defense against oxidative stress), and its downstream target HO-1, were upregulated in samples of bladder cancer (*N* = 16) in comparison to healthy tissue (*N* = 11) (Figure 2). Similarly, factors regulated by hypoxia (HIF-1α and HIF-2α) and their target VEGF were upregulated in bladder cancer samples (Figure 2).

### 3.3. Analysis of miRNA Expression in Cancer Samples

**Figure 3:**
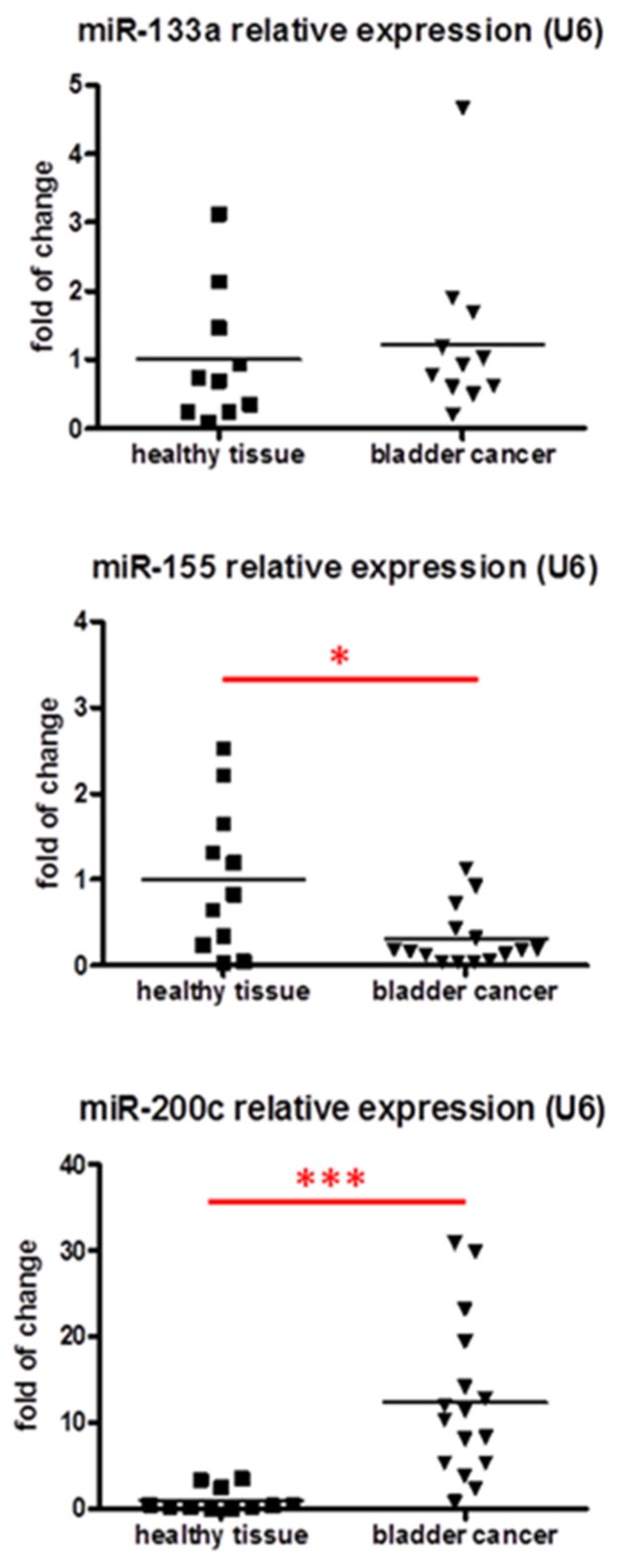
The expression of selected miRNAs in samples of bladder cancer and in healthy tissue. qRT-PCR, *N* = 11–16; each dot represents one individual, line represents a mean; * *p* < 0.05; *** *p* < 0.001.

Analysis of miRNAs showed that the level of miR-200c is significantly induced in samples of bladder cancer, whereas miR-155 is downregulated. No changes were observed in the case of miR-133a (Figure 3).

Spearman’s rank correlation coefficient was used to determine the correlation between miRNAs and the expression of genes analyzed. MiR-200c was found to positively correlate to VEGF expression in bladder cancer (Table 3).

## 4. Discussion

Our results confirm previous data showing the increased production of proinflammatory and proangiogenic cytokines in patients affected by bladder cancer, and suggesting their diagnostic importance [20,54,55,56].

Upregulation of IL-6 was detected in the sera and urine of bladder cancer patients [56,57], as well as in tumors [58,59]. IL-6 was correlated with higher clinical stage, higher recurrence rate, and reduced survival of patients with bladder cancer [58]. TNFα was also detected in the sera and in peripheral blood mononuclear cells of patients with bladder cancer, although no correlation with tumor stages was shown [59,60]. Our results confirm a significantly enhanced level of IL-6 and only a tendency toward such an induction in the case of TNFα.

IL-8 was shown to induce both angiogenesis and tumorigenecity, and in this way it can enhance the metastatic potential of bladder cancer [18,19,20,55,61]. A similar relationship was demonstrated for VEGF expression, which enhances angiogenesis, proliferation, and metastatic potential in urothelial tumors [18,19,20]. Accordingly, we observed the induction of IL-8 (at protein level in sera) and VEGF (at protein level in sera and at mRNA level in the specimen of urothelial bladder cancer).

The upregulation of VEGF correlates to HIF-1α expression. We have also showed for the first time the elevated expression of both Nrf2 and HO-1 in urothelial bladder cancer. However, both Nrf2 and HIFs transcription factors are mostly regulated at protein stability [12,62], though its mRNA increase also indicates possible protein upregulation. It is therefore possible that Nrf2, a known inducer of HO-1 expression in different tissues [22], is also responsible for elevating HO-1 expression in bladder cancer. This supports previous *in vitro* data suggesting that Nrf2 may be associated with the enhanced expression of HO-1 in bladder cancer cells [10], which in turn enhances pathological angiogenesis in tumors and the viability of cells during therapy [10,24,25,26].

The mechanism of proangiogenic, and especially cytoprotective properties of HO-1 in bladder cancer is not well understood. It is associated with increased VEGF, HIF-1α, and HIF-2α [24]. Taking into account that HO-1 also potentiates the expression of other proangiogenic factors [22,63], and that our results show increased IL-8 in the sera of bladder cancer patients, the involvement of other mediators is also possible. HO-1 regulates the cell cycle via the modulation of soluble guanylyl cyclase activity, p38-signalling pathway, or PI3K pathway, which are activated by one of the heme degradation end products, carbon monoxide (CO) [22]. It is therefore possible that these mechanisms might also play a role in bladder cancer. Moreover, in other studies the HO-1 level was shown to correlate with the expression of cyclooxygenase-2 (COX-2) both *in vivo* (in samples of bladder cancer patients [28]) and *in vitro* (in bladder cancer cell lines cultured in hypoxic conditions [24]). COX-2 is a factor associated with carcinogenesis and higher pathological stages of bladder cancer [64]. It requires further analysis to examine the possible link between HO-1 and COX-2 in bladder cancer.

Furthermore, HO-1 is a potent regulator of miRNAs [30], inhibiting among others miR-133a, miR-133b, and miR-1 [30]. The importance of these miRNAs has been suggested in bladder cancer [46,47,48,49,50,51,52,65,66]. However, our analysis does not show the changes in the expression of miR-133a.

In turn, we have observed a decreased expression of miR-155, which was previously shown to be increased in the urine of the patients affected by urothelial bladder cancer [33]. Accordingly, the results showed that miR-155 is upregulated in urothelial tumors and associated with poor survival [67] as well as with the induction of the proliferation of bladder cancer cell line *in vitro* [68]. These results seem contradictory to those presented here; however, it must be noted that the sequence of primers used in that study [67] did not cover the mature miR-155-5p, which is detected in our analysis. MiR-155 was shown to target HIF-1α in murine and human cells [69,70]; therefore, its downregulation may be responsible for the upregulation of HIF-1α observed here in bladder cancer cells. Although miR-155 was demonstrated to target HO-1 in rodent models [71,72] it must be noted that in human cells it upregulates HO-1 expression by targeting Bach1—a repressor of HO-1 transcription [73]. Further studies are necessary to determine if there is any direct link between miR-155 and HO-1 in bladder cancer.

Finally, we have also demonstrated the increased expression of miR-200c in specimens of bladder cancer in comparison to healthy controls, which supports previous data showing an upregulation of this miRNA in bladder cancer [39,40,41]. As miR-200c has so far been shown to target HO-1 and is associated with HO-1 decreased expression in other tissues and cancer types [74,75], we did not expect that it could be a mediator of HO-1 upregulation or action in bladder cancer. On the other hand, miR-200c was positively correlated with VEGF expression in samples of bladder cancer. Similarly, miR-200c overexpression was shown to induce VEGF expression in non-small cell lung cancer [76], although opposite results suggesting that miR-200c directly targets VEGF expression were also obtained for different cancer types [38,77,78]. Therefore it seems that the relationship between miR-200c and VEGF depends on cell type.

In conclusion, we have shown here for the first time that the expression of both HO-1 and Nrf2 is elevated in specimens of bladder cancer. Additionally, HIF-1α and HIF-2α are upregulated at the mRNA level in urothelial bladder cancer and correlate with elevated VEGF expression in tumors and with its increased concentration in the plasma of patients affected by bladder cancer in comparison to healthy controls. The elevated level of proinflammatory and proangiogenic cytokines was also observed in the sera of patients with bladder cancer. Among the miRNAs analyzed, upregulation of miR-200c and downregulation of miR-155 were observed, which may be responsible for the induction of HIF-1α mRNA. The expression of both HO-1 and Nrf2 is increased in bladder cancer compared to healthy tissue.

Further studies with increased number of patients, and the functional assays for the potential targets of microRNAs and transcription factors are necessary to validate the results described here.

## Figures and Tables

**Table 1:** Sequences of starters for genes.

Gene	Sequence of Starters	
EF2	forward	5′-GAC ATC ACC AAG GGT GTG CAG-3′
	reverse	5′-TCA GCA CAC TGG CAT AGA GGC-3′
HO-1	forward	5′-GTG GAG MCG CTT YAC RTA GYG C-3′
	reverse	5′-CTT TCA GAA GGG YCA GGT GWC C-3′
VEGF	forward	5′-ATG CGG ATC AAA CCT CAC CAA GGC-3′
	reverse	5′-TTA ACT CAA GCT GCC TCG CCT TGC-3′
Nrf2	forward	5′-GGG GTA AGA ATA AAG TGG CTG CTC-3′
	reverse	5′-ACA TTG CCA TCT CTT GTT TGC TG-3′
HIF-1α	forward	5′-TGC TTG GTG CTG ATT TGT GA-3′
	reverse	5′-GGT CAG ATG ATC AGA GTC CA-3′
HIF-2α	forward	5′-TCC GAG CAG TGG AGT CAT TCA-3′
	reverse	5′-GTC CAA ATG TGC CGT GTG AAA-3′

**Table 2:** Sequences of starters for miRNA.

miRNA	Sequence Of Specific Starters
U6	5′-CGC AAG GAT GAC ACG CAA ATT C-3′
miRNA-133a	5′-TTG GTC CCC TTC AAC CAG CTG T-3′
miRNA-155	5′-TTA ATG CTA ATT GTG ATA GGG GT-3′
miRNA-200c	5′-TAA TAC TGC CGG GTA ATG ATG GA-3′

**Table 3:** Spearman’s rank correlation of tested miRNA/mRNA; * *p* < 0.05.

	HO-1	Nrf2	VEGF	HIF1	HIF2
miR-133a	−0.329	−0.347	0.161	0.063	0.109
miR-155	0.194	−0.151	−0.009	0.411	−0.385
miR-200c	0.259	0.406	0.606 *	0.365	0.424
